# The Coming of Peace and Its Awakening Powers

**Published:** 1919-07-12

**Authors:** 


### The Hospital

The Workers' Newspaper of Administrative Medicine and Institutional Life, Administration, National Insurance and Health.

No. 1727, Vol. LXVI. SATURDAY, JULY 12, 1919. PRICE TWOPENCE

### THE COMING OF PEACE AND ITS AWAKENING POWERS.

The national atmosphere throughout the British Islands is daily exhibiting with appreciable distinctness more and more evidence of the arrival of Peace in our midst. The splendid reception and welcome given to the soldier-men of London, who had returned from the war, some of them demobilised and in civilian dress, some of them in the uniforms of the various regiments and services in which they serve, most of them the sons, and all of them the fellow-workers of the inhabitants of the Metropolis of the Empire, gave evidence of this atmosphere of peace with touching force. The march of these soldiers, the manner of their coming, the welcome they received, and the whole atmosphere of London from end to end on July 5, 1919, which will properly be acclaimed amongst us as Victory Saturday, must have stirred the hearts and feelings of all who witnessed it. Who of us all who were able suffered him or herself or the joyous children to be absent from this spontaneous outpouring of joyous recognition and heart-felt thanks to the brave fellows who gave up their occupations to defend the Motherland, and proved themselves throughout the war to be warriors of the strenuous type who put their hearts into the work and faced the awful dangers and happenings resulting from the vigorous conduct of the worst war the world has ever seen? It is good for us Men and Women, Fathers and Mothers, Londoners of all classes and types, to acclaim and cheer with heart and soul our brave fellow-citizens who risked their lives, and many of them have forfeited, to maintain the honour and security of the Homeland. It proved a great day and a great occasion, and the people, like the sailors and soldiers and airmen, rose to the occasion and made it memorable for all time. Then came Sunday, Peace Sunday, the Day of Thanksgiving and Gratitude to Almighty God for the gracious protection He has extended to the Allies, and The Victory which the signing of Peace brings to the understanding and recognition of the least observant of mankind.

The great services at St. Paul's Cathedral, and especially the assembly in tens of thousands of The People, at its Western doors, where a special service was held, called forth an outpouring of thanksgiving to Almighty God, and of loyalty and gratitude, which were made strikingly beautiful by the raising of a multitude of voices in harmonious acclaim, expressing heartfelt gratitude and The Faith which so stirred the hearts of all present that the singing penetrated the mind and uplifted the soul of all those who heard it. Who, on their return from the services, could there be who was not imbued with the feeling that there is no happiness so real and uplifting for the human race as the arousing of their spiritual life, its awakening and articulation? The service in Trafalgar Square was a wonderful outpouring of Spirit too, and will live in the memory of those who attended it so long as life endures. Everywhere throughout the country, not one Church but all Churches, not one creed but all creeds, united in thanksgiving services which were not confined to Trafalgar Square, but took place wherever throughout Great Britain there was a House of God, and Men and Women to fill it.

Yes, Peace Sunday, July 6, is something to be thankful for. It gave vitalising evidence of the recognition of Almighty God, and man's dependence upon Him. It opened the eyes of Preachers and Teachers, Bishops and Clergy, Ministers, Priests and People to the created's dependence upon the Creator, and the bountiful goodness of Almighty God, the Father of us all. Is this great uplifting of the soul to be prevented continuous and public expression by Men, Women and Children in these islands through conventional methods, perfunctory ministrations, and the absence of evidence that Every Man of God, from the Archbishop to the humblest attendant in our places of worship, **believes in the living God, acknowledges his dependence upon our Heavenly Father, and looks to Him with thankfulness and praise for the continued blessings which we all enjoy? Let the sluggards wake up, or at least awaken to their unworthiness, and the conventional atmosphere which they so often too frequently create. Do they forget the sheep, and have they ceased to be faithful shepherds of God's people, wherever they may minister? Take on the atmosphere of God's House and bear in mind His continual presence therein.**

